# Dissociation, trauma, and borderline personality disorder

**DOI:** 10.1186/s40479-022-00184-y

**Published:** 2022-04-19

**Authors:** Annegret Krause-Utz

**Affiliations:** 1 Department of Clinical Psychology, Institute of Psychology, Leiden University, Leiden, The Netherlands; 2 Leiden Institute for Brain and Cognition, Leiden University, Leiden, The Netherlands

**Keywords:** Borderline personality disorder, Dissociation, Trauma, Post-traumatic stress disorder

## Abstract

Dissociation is a complex phenomenon, which occurs in various clinical conditions, including dissociative disorders, (complex) post-traumatic stress disorder (CPTSD, PTSD), and borderline personality disorder (BPD). Traumatic stress is considered an important risk factor, while the etiology of dissociation is still debated. Next to traumatic experiences, temperamental and neurobiological vulnerabilities seem to contribute to the development of dissociation. Stress-related dissociation is a prevalent symptom of BPD, which may interfere with psychosocial functioning and treatment outcome. More research in the field is strongly needed to improve the understanding and management of this complex phenomenon. This article collection brings together research on dissociation and trauma, with a special focus on BPD or sub-clinical expressions of BPD. In this editorial, recent conceptualizations of dissociation and relevant previous research are introduced in order to provide a framework for this novel research.

Dissociation is a complex trans-diagnostic phenomenon, which comprises a wide range of symptoms [1, 2]. It is broadly defined as a discontinuity or disruption of usually integrated functions, such as consciousness, perception, attention, memory, and identity [1, 3]. Psychological symptoms include subjective detachment from the own person (depersonalization) or the environment (derealization), which may be perceived as unreal, blurry, movie-like, or lacking significance. Memory disruptions can range from a diminished ability to access normally amenable information to dissociative amnesia [4]. Somatoform symptoms include altered pain perception (analgesia) and a loss of voluntary motor control [3]. Dissociative experiences exist on a continuum and also occur in non-clinical populations [5]. In clinical settings, dissociation is a core symptom of various disorders, including dissociative disorders (e.g., dissociative identity disorder, DID), (complex) post-traumatic stress disorder (CPTSD, PTSD), and borderline personality disorder (BPD) [2]. Dissociative symptoms may further occur in schizophrenia [6], major depressive disorder [7], bipolar disorder [8] and obsessive-compulsive disorder [9]. The differentiation between dissociative and psychotic symptoms can be challenging [10–12]. Many patients who experience pathological dissociation report a long history of hospitalizations and misdiagnoses, before finding adequate treatment [13, 14]. Therefore, it remains of upmost importance to further improve the understanding and management of pathological dissociation [15, 16].

## Etiological models of dissociation

The etiology of dissociation is still strongly debated. Most models propose a complex interplay of multiple factors, including genetic neurobiological vulnerabilities, temperamental dispositions, and environmental factors [1, 3]. Currently, two main perspectives exist: trauma models and socio-cognitive models.

Trauma models consider psychological trauma a crucial risk factor in the development of dissociation [17–21]. It has been proposed that dissociation may serve as an (evolutionary-based) defense mechanism to cope with unbearable, overwhelming experiences during a potentially traumatizing event [4, 22, 23]. This initially adaptive response may become maladaptive if generalized to other stressful situations. Dissociation can hinder the integration of emotions, thoughts, and sensations [24]. Salient characteristics of a stressful event may be stored (compartmentalized) as fragmented memories, which can later recur as intrusive flashbacks [4]. Dissociation can also interfere with emotional learning and hinder the acquisition of new information in stressful contexts, e.g., during exposure therapy [25–27]. Numerous studies have provided empirical evidence for a link between dissociation and psycho-trauma, including severe childhood maltreatment [18, 19, 28–34]. A recent meta-analysis found strong associations between dissociation and emotional, sexual, and physical abuse by caregivers. Earlier age of onset, longer duration of abuse, and parental abuse predicted more severe dissociation [28].

Other researchers point out that trauma is neither a necessary nor sufficient factor for the development of dissociation and question the direct causal link between the two. Socio-cognitive models emphasize the role of cognitive predispositions (e.g., fantasy-proneness, suggestibility, hyper-associativity) social factors (e.g., media influences, questioning techniques) [34, 35] and sleep disturbances [36]. These variables may contribute to the way individuals, who are prone to dissociation, perceive stressful events and express emotional experiences and inconsistencies in identity [37].

Up to now, the etiology of dissociation remains elusive and different views co-exist.

## Potential neurobiological mechanisms of dissociation

A growing number of studies have investigated potential neurobiological underpinnings of dissociation, which are not yet fully understood. A recent systematic review of 205 neuroimaging studies suggests that enhanced task-related activity of the inferior frontal gyrus and medial prefrontal cortex may be linked to dissociation [38]. Largely in line with this, another systematic review concluded that functional alterations in frontal regions are most consistently observed in individuals with dissociative symptoms [39]. This may point to an increased recruitment of brain regions implicated in arousal modulation [21, 40]. Further evidence for this idea stems from studies that used script-driven imagery to induce acute dissociative symptoms and study their impact on information processing. Patients with acute dissociation after script-driven imagery showed increased activity in the inferior frontal gyrus during an inhibitory task [25, 26].

With respect to brain structure, decreased volumes in the hippocampus, basal ganglia, and thalamus were most consistently correlated to dissociative symptoms [38]. However, findings are quite diverse and replication studies are strongly needed [39].

With regards to psychophysiological research, findings are mixed [38]. Some studies suggest that dissociation may be accompanied by changes in heart rate (variability), skin conductance response (SCR), and fear-potentiated startle responses [21, 40]. Individuals with acute dissociation showed reduced startle response [41–43], diminished SCR [42, 44] and a decline in heart rate [45]. However, contradictory findings have also been reported [38, 46].

## Dissociation in Borderline Personality Disorder (BPD)

The effects of dissociation on psychosocial functioning may depend on the larger psychopathological context, e.g., emotional dysregulation and identity problems [47, 48]. In BPD, stress-related dissociation is a core symptom, closely linked to other features of the disorder [1, 49]. Up to 80% of patients with BPD report transient dissociative symptoms, such as derealization, depersonalization, numbing, and analgesia [1, 50]. The severity of dissociation is correlated to the severity of traumatic experiences [23, 28, 29]. While trauma is an important risk factor for the etiology of BPD in individuals with genetic, temperamental neurobiological vulnerabilities [51–55], non-trauma related pathways are also involved [56, 57]. Dissociation in BPD is closely linked to emotion dysregulation, disturbed identity, and relationship problems.

*Emotion dysregulation* includes a tendency to experience intense overwhelming emotions. The strength, frequency and intensity of emotional distress was found to increase and decrease along with dissociative symptoms [58]. Dissociation may exaggerate difficulties identifying emotions [59]. During emotional distress, patients with BPD show impulsive decision making [60] and often use maladaptive strategies to regulate their emotions, e.g., non-suicidal self-injury (NSSI) [61, 62]. Terminating states of dissociation can be a strong motive for NSSI [63, 64]. A recent study suggests that dissociation is linked to pain hyposensitivity in patients with acute BPD, but not in those who show remission [65].

A novel line of research further suggests that dissociation is linked to reduced body ownership, i.e., the certainty that body parts belong to oneself [66]. In a recent study, female patients with BPD reported significantly lower levels of body awareness and significantly higher levels of body dissociation compared to healthy women. Significant positive correlations between body dissociation, traumatic childhood experiences, and emotion regulation were found [67].

*Identity disturbances* are another core domain of BPD. Individuals with the disorder experience rapid changes in self-image and perceived their identity as incoherent, inconsistent, vague, or fragmented [68–70]. They also report chronic feelings of emptiness [71]. Sense of self strongly depends on current self-esteem, which is highly unstable under daily life condition [69, 72]. Identity disturbances in BPD show considerable overlap with dissociative symptoms and may be hard to distinguish [73].

*Interpersonal disturbances* in BPD involve profound mistrust, rejection hypersensitivity, and strong ambivalence between a need for closeness and a need for autonomy [74–77]. It is crucial to improve the understanding of dissociation in this context. In intimate relationships, dissociation may reduce assertiveness and lead to a subjective detachment from violent behaviour [78, 79]. There is preliminary evidence that dissociation contributes to sexual revictimization after child sexual abuse, when BPD symptoms and emotion regulation are taken into account [80]. However, much more prospective research is needed to understand how dissociation interferes with interpersonal functioning in BPD. For instance, future studies may investigate how dissociation interferes with intimacy, trust, and the processing of both positive and negative experiences in close relationships.

## Methodological challenges and outstanding research questions

Research on dissociation is rapidly increasing, which has considerably improved its understanding. At the same time, the use of different conceptualizations and methodologies hinders the integration and comparison of these findings.

Acute dissociative states should be differentiated from dissociation proneness, i.e., the general tendency to experience dissociation [5, 81]. For both concepts, various validated measures exist, such as the Dissociative Experience Scale (DES, trait dissociation) [82] Dissociation Stress Scale (DSS, state dissociation [83] or the Structured Clinical Interview for DSM-IV Dissociative Disorders (SCID-D) [84]). Script-driven imagery may be used to induce acute states of stress [45, 65] and dissociation [25, 26, 85]. In this paradigm, a personal narrative of an autobiographical situation is created. While listening to the script, participants are asked to recall the autobiographical situation as vividly as possible. When combined with other measures (e.g., behavioral, neuropsychological, psychophysiological, neuroimaging outcomes), direct effects of acute dissociation can be studied. These effects may differ from alterations associated with trait dissociation. Sample characteristics (e.g., comorbidities, trauma histories, medication status) may further hinder a straight-forward comparison of results. There is overlap of BPD with complex presentations of PTSD, following repeated interpersonal trauma [86, 87]. A recent study suggests that traumatized women who reported more dissociative symptoms showed more symptoms of both complex PTSD and BPD, as compared to distinct symptom profiles of CPTSD, PTSD, or BPD [88]. More research is needed to investigate how these symptom profiles can be distinguished [89].

## Possible clinical implications

Dissociation can have an impact on treatment, which was most consistently shown for BPD. For PTSD without BPD this may not be the case, according to meta-analytical evidence [90]. In BPD, dissociative symptoms predicted poor response to psychodynamic therapy [91, 92]. More severe dissociation further predicted negative treatment outcome of Dialectical Behaviour Therapy (DBT) in two independent studies [93, 94]. A multilevel meta-analysis of different psychotherapies for BPD suggests that changes in dissociative symptomatology may be harder to achieve, as compared to symptoms of affective instability and overall BPD severity [95].

At the same time, there is evidence that dissociative symptoms can be successfully targeted during treatment. For instance, an adapted version of DBT for patients with BPD and PTSD, which involves constant monitoring and management of dissociation, resulted in better treatment outcomes, compared to standard DBT, standard Cognitive Processing Therapy, and control treatment [15, 96–99]. Evidence-based treatments for BPD, such as DBT, Mentalization-Based Treatment (MBT), Schema-Focused Therapy (SFT), and Transference-Focused Psychotherapy (TFP) target emotion dysregulation and identity problems. Thereby, they may either directly or indirectly improve dissociation [100, 101]. In a prospective follow-up study over 20 years, a decrease of depersonalization and derealization symptoms was strongly associated with overall BPD recovery status [102].

## Conclusion

Overall, the understanding of dissociation and its link to trauma and BPD is steadily increasing. Since dissociation comprises various symptoms and occurs in different psychopathological contexts, a careful assessment of symptoms may help to further deepen this knowledge. In BPD, dissociation is closely linked to other symptoms, such as emotion dysregulation, disrupted identity, and interpersonal disturbances. This may interfere with affective-cognitive functioning (e.g., interference inhibition), body perception, and treatment. Neurobiological findings are still diverse but hint towards increased activity in frontal regions (e.g., inferior frontal gyrus) and temporal areas during dissociative states. Differences in the definition and assessment of dissociation as well as sample characteristics (e.g., comorbidities, trauma history) hinder a straightforward interpretation and comparison of results. Given this complexity, more research, including longitudinal designs with multiple measures, is strongly needed.

## Data Availability

N.A.
